# Comparison of Performance of Serum and Plasma in Panbio Dengue and Japanese Encephalitis Virus Enzyme-Linked Immunosorbent Assays

**DOI:** 10.4269/ajtmh.2012.12-0043

**Published:** 2012-09-05

**Authors:** Stuart D. Blacksell, Sue J. Lee, Anisone Chanthongthip, Thaksinaporn Taojaikong, Soulignasack Thongpaseuth, Tanja Hübscher, Paul N. Newton

**Affiliations:** Lao-Oxford-Mahosot Hospital-Wellcome Trust Research Unit, Microbiology Laboratory, Mahosot Hospital, Vientiane, Laos; Mahidol University-Oxford Tropical Medicine Research Unit, Faculty of Tropical Medicine, Mahidol University, Bangkok, Thailand; Centre for Clinical Vaccinology and Tropical Medicine, University of Oxford, Churchill Hospital, Oxford, United Kingdom; Travel and Tropical Medicine and General Medicine, Hohmad Clinic, Thun, Switzerland

## Abstract

We examined the comparative performance of serum and plasma (in dipotassium EDTA) in Panbio Dengue enzyme-linked immunosorbent assays (ELISAs) for detection of non-structural protein 1 (NS1), IgM, and IgG, and a dengue/Japanese encephalitis virus (JEV) combination IgM ELISA in a prospective series of 201 patients with suspected dengue in Laos. Paired comparisons of medians from serum and plasma samples were not significantly different for Dengue IgM, and NS1 which had the highest number of discordant pairs (both 2%; *P* = 0.13 and *P* = 0.25, respectively). Comparison of qualitative final diagnostic interpretations for serum and plasma samples were not significantly different: only 1.5% (3 of 201 for Dengue/JEV IgM and Dengue IgG) and 2.0% (4 of 201; IgM and NS1) showed discordant pairs. These results demonstrate that plasma containing EDTA is suitable for use in these ELISAs.

Manufacturers of diagnostic assays make specific recommendations for the sample matrix to be used for testing because blood preservatives or anticoagulants may affect assay performance. Serum centrifuged from clotted blood is often the sample of choice because it contains no chemical additives. Instructions for many commercial enzyme-linked immunosorbent assays (ELISAs) and rapid tests for diagnosis of acute dengue and Japanese encephalitis virus (JEV) infections do not state whether plasma may be used and, if so, which anticoagulant agents, such as lithium heparin, sodium fluoride, potassium oxalate, or EDTA, are appropriate.

We have therefore examined the comparative performance of paired serum and plasma samples of four well-established and previously assessed Panbio ELISAs (Alere, Brisbane, Queensland, Australia) for detection of dengue virus non-structural protein 1 (NS1),^1^ IgM,^2^ IgG,^2^ and a JEV IgM^3^ ELISA. These kits state that the test should be performed on serum only and that the use of whole blood, plasma, or other specimen matrix has not been established.

Samples (n = 201) were prospectively collected from all patients with suspected dengue-like or JEV-like illness at Mahosot Hospital, Vientiane, Laos during August–November 2010. Ethical clearance was provided by the Ethical Review Committee of the Faculty of Medical Sciences, National University of Laos (Vientiane, Laos) and the Oxford University Tropical Ethics Research Committee (Oxford, United Kingdom).

After informed written consent was obtained, patients were admitted to the study if the responsible physician diagnosed suspected dengue, defined as an acute febrile illness with ≥ 2 of the following features: headache, retro-orbital pain, myalgia, arthralgia, rash, hemorrhagic manifestations, or leukopenia according to World Health Organization guidelines.^4^ Venous blood samples were collected on the day of admission (admission specimen) and on the day of discharge from hospital (convalescent specimen). Serum was prepared by centrifugation of 5 mL of whole blood that was collected into plain 5-mL polystyrene blood collection tubes sterilized with gamma irradiation (Z6744; Teklab, Sacriston, United Kingdom), allowed to clot, and then centrifuged at 2,000 × *g* for 10 minutes. Plasma was prepared by centrifugation, as for serum, from 5 mL whole blood collected into 5-mL blood collection tubes containing 1.75 mg of dipotassium EDTA/mL (catalog no. K6740; Teklab). The two sample types were taken from the same blood draw with the same syringe and stored in the same –80°C freezers until ELISAs were performed.

The assays assessed were the Panbio Dengue Early NS1 antigen (catalog no. E-DEN01P second generation; Alere), Panbio Dengue IgM capture (catalog no. E-DEN01M; Alere), Dengue IgG capture (catalog no. DEN02G; Alere), and Panbio Japanese Encephalitis/Dengue IgM combo (catalog no. E-JED01C; Alere) ELISAs. Serum and plasma samples were tested in duplicate on the same ELISA plate to minimize variation. All assays were performed according to the manufacturer's instructions and results (Panbio Units) and final interpretations were calculated (i.e., dengue or JEV positive, negative, or inconclusive) as per the prescribed method. Inconclusive results were considered negative.

Quantitative (Panbio units) and qualitative results (positive or negative) for paired serum and plasma samples for each ELISA were compared by using STATA version 10.0 (StataCorp LP, College Station, TX). The Wilcoxon signed-rank test for matched pairs was used to test equality of Panbio Units for each ELISA. Differences in qualitative results for final assay interpretation were assessed by using McNemar's chi-square test. The range within which one would expect 95% of the values from the paired samples to lie (i.e., limits of agreement) were calculated by using the Bland-Altman method for each ELISA.^5^,^6^ *P* values < 0.05 were considered significant.

Comparison of the Panbio unit values and of final interpretations (positive or negative using manufacturer's criteria) for all ELISAs (Table 1) demonstrated no significant differences, with the exception of the JEV/Dengue Combo IgM ELISA, which showed significantly different results for plasma and serum for the Panbio unit comparison (*P* = 0.02) but not for the final interpretation (*P* = 0.5). There were 1.5% (3 of 201) discordant pairs for the IgG capture ELISA and 2.0% (4 of 201) discordant pairs for the IgM capture and NS1 antigen ELISAs (Table 1). Mean differences for serum and plasma Panbio units was generally small ranging from 0.07 (JEV Combo IgM capture ELISA) to 1.0 (JEV/Dengue Combo IgM capture ELISA) (Table 1). Bland and Altman 95% limits of agreement ranged from −9.50 to 9.74 for the IgG capture ELISA to –15.67 to 17.67 for the JEV/Dengue Combo IgM capture ELISA) (Table 1 and Figure 1). Comparison of Panbio unit results for dengue-positive and dengue-negative samples as determined by using the assay interpretation criteria (Table 2 and Figure 2). demonstrated that with the exception of the JEV/Dengue Combo IgM capture ELISA (*P* = 0.03), there were no significant differences.

**Figure 1.**
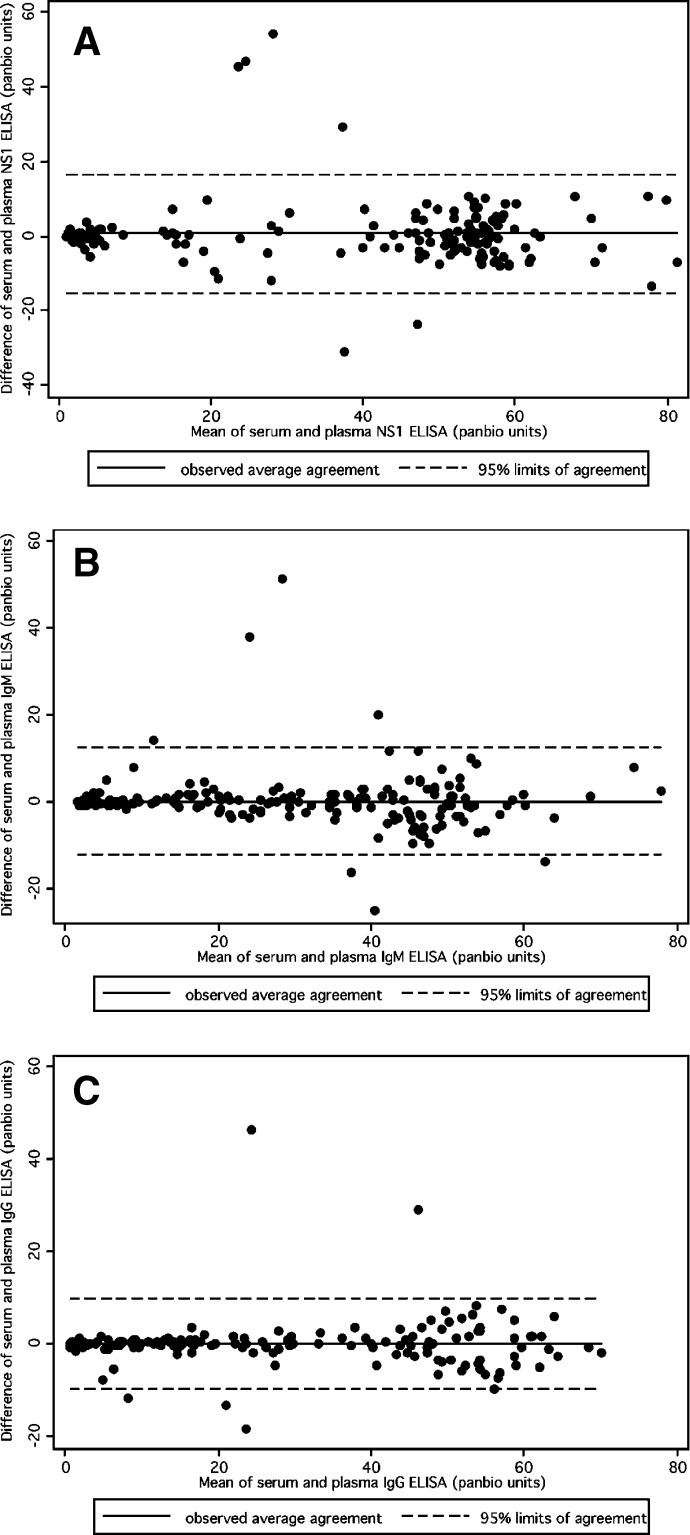
Difference against mean plot for plasma and serum Panbio units for (**A**) nonstructural protein 1 (NS1), (**B**) IgM, and (**C**) IgG enzyme-linked immunosorbent assays (ELISAs). Bland and Altman 95% limits of agreement (**dashed lines**) and mean (**solid lines**) are indicated.

**Figure 2.**
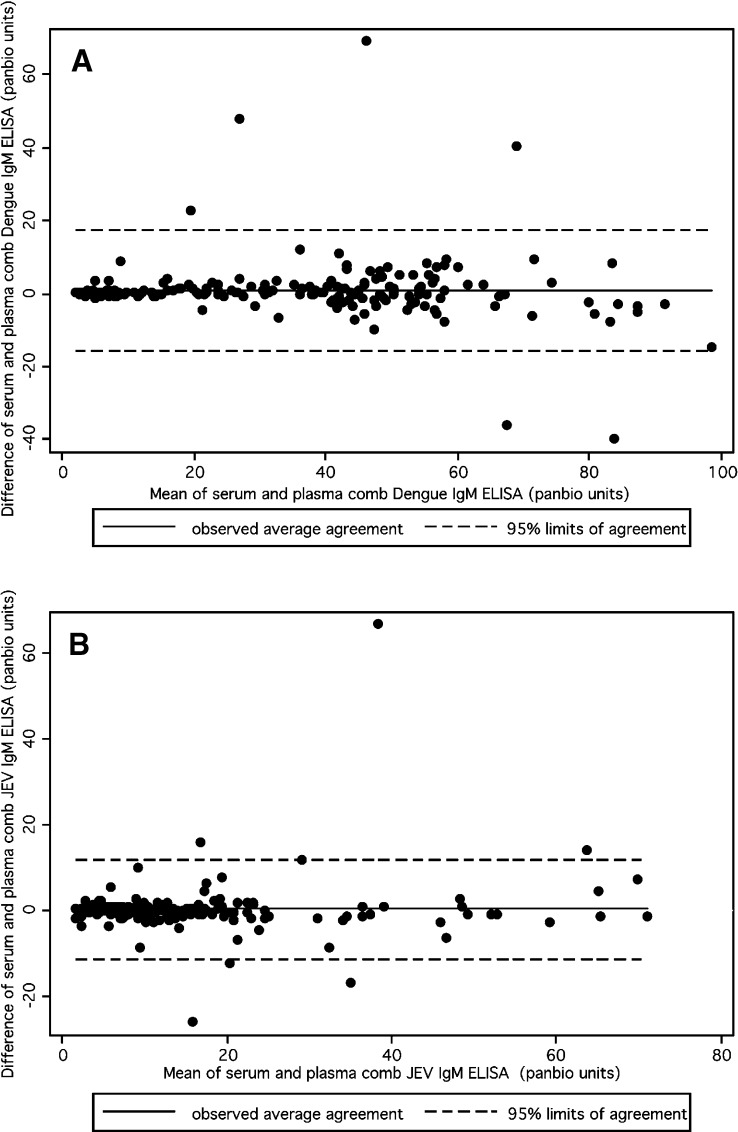
Difference against mean plot for plasma and serum Panbio units for combination (**A**) dengue and (**B**) Japanese encephalitis virus (JEV) IgM enzyme-linked immunosorbent assays (ELISAs). Bland and Altman 95% limits of agreement (**dashed lines**) and mean (**solid lines**) are indicated.

These results suggest that plasma is a suitable sample for use in Panbio dengue and JEV ELISAs. Other studies with immunoassays for C-reactive protein,^7^ N-terminal pro-brain natriuretic peptide,^8^ and cryptococcal antigen^9^ have also demonstrated that results from serum and plasma samples are comparable. Other factors that can potentially affect ELISA results are temperature storage conditions and repeated freeze–thaw cycles. However, these affects appear to be less pronounced for ELISA detection of antibodies and viral antigens.^10^–^12^ Further studies are required to determine the affect of other anticoagulant agents such as lithium heparin, sodium fluoride and potassium oxalate, as well as the affect of sample timing, on anticoagulant-treated samples on assay performance.

## Figures and Tables

**Table 1:** Quantitative (Panbio units) results for comparison of 201 paired serum and plasma samples tested in Panbio dengue/JEV ELISAs*

ELISA	Target	Sample	Overall		Difference		Final interpretation†	
			Median Panbio units (IQR)	Wilcoxon signed-rank value (*P*)	Mean (95% CI)	Bland and Altman 95% limits of agreement	Discordant pairs, no. (%)	McNemar's χ^2^ (*P*)
Dengue/JEV IgM	Dengue IgM	Serum	36.47 (8.82–50.84)	2.34 (0.02)	1.0 (–0.18 to 2.18)	−15.67 to 17.67	3 (1.5)	2.0 (0.5)
		Plasma	31.46 (8.19–49.4)					
	JEV IgM	Serum	8.70 (5.38–17.31)	−1.62 (0.11)	0.07 (–0.73 to 0.89)	−11.51 to 11.65	0	0
		Plasma	8.90 (5.08–16.32)					
IgG capture	Dengue IgG	Serum	14.51 (2.56–45.15)	0.76 (0.45)	0.12 (–0.56 to 0.80)	−9.50 to 9.74	3 (1.5)	0.0 (1.0)
		Plasma	14.78 (2.69–44.6)					
IgM capture	Dengue IgM	Serum	27.81 (7.49–44.87)	0.61 (0.54)	0.39 (–0.48 to 1.25)	−11.82 to 12.59	4 (2.0)	4.0 (0.13)
		Plasma	26.72 (6.44–46.72)					
NS1	Dengue NS1	Serum	43.71 (2.06–54.27)	0.33 (0.74)	0.61 (–0.51 to −1.72)	−15.12 to 16.33	4 (2.0)	3.0 (0.25)
		Plasma	41.57 (1.97–54.52)					

* JEV = Japanese encephalitis virus; ELISA = enzyme-linked immunosorbent assay; IQR = interquartile range; CI = confidence interval; NS1 = non-structural protein 1.

† Using Panbio criteria.

**Table 2:** Comparison of Panbio units results for 201 dengue-positive and dengue-negative serum and plasma samples tested in Panbio dengue/JEV ELISAs*

ELISA	Infection	Dengue status†	No.	Wilcoxon signed-rank *P*
Dengue/JEV IgM	Dengue	Positive	148	0.03
		Negative	53	0.38
	JEV	Positive	148	0.14
		Negative	53	0.42
IgG capture		Positive	83	0.79
		Negative	118	0.20
IgM capture		Positive	142	0.91
		Negative	59	0.38
NS1		Positive	128	0.73
		Negative	73	0.95

* JEV = Japanese encephalitis virus; ELISA = enzyme-linked immunosorbent assay; range; NS1 = non-structural protein 1.

† Using Panbio criteria for serum samples.
